# Follow-up patterns after positive primary aldosteronism screening

**DOI:** 10.1371/journal.pone.0333534

**Published:** 2025-10-23

**Authors:** Raul Herrera, Michael Salim, Meng Xu, Sisi Ma, Jacob Kohlenberg, Kidmealem Zekarias

**Affiliations:** 1 Division of Diabetes, Endocrinology and Metabolism, Department of Medicine, University of Minnesota, Minneapolis, Minnesota, United States of America; 2 Department of Health Informatics, Institute for Health Informatics, University of Minnesota, Minneapolis, Minnesota, United States of America; Stellenbosch University Faculty of Medicine and Health Sciences, SOUTH AFRICA

## Abstract

Primary aldosteronism (PA) screening remains underdiagnosed at <2% of eligible patients, with inconsistent interpretation of positive screening results across institutions. This study investigated whether patients with positive PA case screening test results received appropriate follow-up interventions. We assessed plasma renin activity (PRA) and aldosterone concentration (PAC), calculating the aldosterone-to-renin ratio (ARR). Three commonly used criteria defined positive case detection: PAC ≥ 10 ng/dL and PRA < 1 ng/mL/h; ARR ≥ 20 and PAC ≥ 10 ng/dL; and ARR ≥ 30 and PAC ≥ 10 ng/dL. We identified 237 patients meeting at least one criterion between April 2018 and May 2023, then assessed follow-up care at least 6 months post-screening. Adequate follow-up included documented abnormal results in progress notes, further PA diagnostics, new MRA treatment, or specialist referral. Inadequate follow-up was observed across all screening criteria: 37% (82/222) of patients meeting PAC ≥ 10 ng/dL and PRA < 1 ng/mL/h, 38% (89/237) of those meeting ARR ≥ 20 and PAC ≥ 10 ng/dL, and 30% (53/180) of those meeting ARR ≥ 30 and PAC ≥ 10 ng/dL lacked appropriate care. History of hypokalemia (p < 0.001), English as primary language (p ≤ 0.007), and higher socioeconomic status (p ≤ 0.06) were consistently associated with adequate follow-up. These findings highlight the need for standardized criteria and automated alerts to improve follow-up after positive PA screening.

## Introduction

Primary aldosteronism (PA) has been increasingly recognized as one of the most common causes of secondary hypertension [1–4]. The reported prevalence of PA varies widely in the primary care setting (3.2–12.7%) and in referral centers (1.0–29.8%) [1–4]. These estimates likely underestimate the true burden of PA, as there continues to be a lack of systematic screening for PA [5,6]. Following case detection, treatment is essential, as untreated PA is associated with a higher risk of cardiovascular, kidney, and metabolic complications [7–11].

The clinical importance of identifying PA extends beyond blood pressure control. Patients with PA have significantly higher cardiovascular morbidity compared to those with essential hypertension, including increased risks of myocardial infarction, stroke, and heart failure [9]. However, the diagnostic pathway for PA is complex, typically requiring positive screening results to trigger confirmatory testing, imaging studies, and subspecialty evaluation then treatment.

During our study period, there was considerable variability in institutional criteria for interpreting positive screening results, with different aldosterone-to-renin ratio and plasma aldosterone concentration thresholds used across centers [12]. This lack of standardized interpretation may contribute to inconsistent recognition and follow-up of abnormal screening results, potentially delaying diagnosis and treatment.

To address inadequate PA screening, quality improvement initiatives have been tried and appear to be promising [13,14]. However, even if screening rates increase, improved outcomes cannot be achieved unless positive case detection results are recognized and acted upon. Despite the established importance of timely PA diagnosis, to our knowledge, no studies have systematically examined the recognition of positive PA case detection and the adequacy of subsequent follow-up interventions. The objectives of this study were to: (1) determine the frequency of adequate follow-up after positive PA screening across different screening criteria and (2) identify factors associated with adequate versus inadequate follow-up. Given the absence of clear consensus on PA screening criteria and the complexity of the diagnostic pathway, we hypothesized that follow-up after positive screening would be suboptimal and variable.

## Materials and methods

### Ethics approval

This study was approved by the University of Minnesota Institutional Review Board (IRB) [STUDY00018742]. Due to the retrospective nature of this study using de-identified electronic health record data, the IRB waived the requirement for informed consent. All procedures were performed in accordance with institutional guidelines and regulations.

### Study data and population

We conducted a retrospective cohort study of adult patients with history of hypertension who underwent biochemical evaluation for PA within an Academic health center in the Midwest United States. We extracted data for all patients who had both plasma aldosterone concentration and plasma renin activity measured between April 2018 and May 2023. Data were accessed for research purposes between January 2024 and September 2024. Authors had access to information that could identify individual participants during data collection but not after data collection, as all data were de-identified following extraction. Given the retrospective design using electronic health record data, we were unable to determine the specific reason for the initial clinical evaluation or laboratory testing (e.g., routine hypertension management, new-onset hypertension, resistant hypertension, or targeted PA screening). Our study focused exclusively on analyzing follow-up patterns after positive screening results regardless of the initial clinical context that prompted testing. In our institution, aldosterone is measured using the Liaison XL chemiluminescent immunoassay and renin activity is measured with a quantitative enzyme-linked immunosorbent assay (ARUP laboratory). The institutional reference range for aldosterone-to-renin ratio is 0.0–25.0. Blood samples are recommended to be collected mid-morning after patients had been sitting, standing, or walking for at least 2 hours and seated for 5–15 minutes prior to collection. Aldosterone samples were collected in red-top or gold-top (gel) tubes, while renin samples were collected in purple EDTA tubes. Samples were separated from cells within 2 hours of collection, with plasma/serum stored frozen until analysis. Renin samples were processed and reported within 5 days, while aldosterone was performed and reported twice weekly. We included patients who underwent PA screening between April 2018 and May 2023. During the study period, our institution’s laboratory provided reference ranges for individual plasma aldosterone concentration (PAC) and plasma renin activity (PRA) measurements but did not establish a specific cutoff value for the aldosterone-to-renin ratio (ARR) or provide automated alerts for elevated ARR values. Our institution’s electronic health record system requires providers to acknowledge laboratory results but does not include automated alerts specifically for abnormal aldosterone-to-renin ratio values or other PA screening parameters during the study period. Provider notification of results relied on standard result review workflows rather than automated alerting for PA-specific abnormalities. Following completion of this study, our institution implemented automated alerts for abnormal PA screening results to address the follow-up gaps identified in this research.

To account for variability in clinical practice and different diagnostic thresholds cited in the literature, we evaluated follow-up patterns using three commonly referenced criteria for positive case detection: (1) PAC ≥ 10 ng/dL with PRA < 1 ng/mL/h; (2) ARR ≥ 20 with PAC ≥ 10 ng/dL; and (3) ARR ≥ 30 with PAC ≥ 10 ng/dL. These criteria were based on established diagnostic thresholds from the Endocrine Society guidelines and commonly used criteria in prevalence studies [1,3–5,12]. These values were calculated by research staff during data analysis and were not flagged in the electronic health record at the time of testing. Inclusion criteria required participants to be 18 years or older and have a diagnosis of hypertension. Exclusion criteria included patients without a documented diagnosis of hypertension (to exclude those who might have had aldosterone labs checked for different reasons such as adrenal incidentalomas or other non-hypertensive indications). Patients were included if they had a positive PA screen based on at least one of three established diagnostic criteria: (1) PAC ≥ 10 ng/dL and PRA < 1 ng/mL/h, (2) ARR ≥ 20 and PAC ≥ 10 ng/dL, or (3) ARR ≥ 30 and PAC ≥ 10 ng/dL. These criteria were applied retrospectively by the research team during data analysis.

The following information was extracted from the electronic health record (EHR): demographic data, medications, comorbidities based on the International Classification of Diseases, 9th and 10th Revisions (ICD-9/ICD-10), vital signs, and laboratory data. Uncontrolled blood pressure was defined as systolic blood pressure ≥130 mmHg or diastolic blood pressure ≥80 mmHg on 2 of the 3 most recent ambulatory measurements. Body mass index (BMI) was categorized into underweight (<18.5 kg/m2), normal (18.5–24.9 kg/m2), overweight (25.0–29.9 kg/m2), and obese (≥30 kg/m2) based on the most recent weight and height measurements. Cardiovascular comorbidities were assessed, including history of myocardial infarction, peripheral arterial disease, ischemic heart disease, and cerebrovascular events (both transient ischemic attacks and strokes). We classified chronic kidney disease (CKD) severity as mild (stages 1, 2, or 3a) or severe (stages 3b, 4, or 5, including patients on dialysis). Socioeconomic status was categorized using income levels defined as: 1 = Extremely Low Income (≤ $27,767); 2 = Very Low Income ($28,692 - $46,278); 3 = Low Income ($47,204 - $74,045); 4 = Moderate Income ($74,971 - $111,067). These categories are derived from institutional classification systems such as the U.S. Department of Housing and Urban Development (HUD) income limits and federal poverty guidelines, which stratify socioeconomic status by absolute and relative income thresholds [15]. This approach enables alignment with federal policy standards and facilitates examination of gradients in health outcomes across distinct SES strata, as recommended in epidemiologic studies [15–17].

### Outcome measures and statistical analysis

Samples were divided further into 3 positive case detection categories based on the criteria they met. A manual EHR review was performed for all eligible patients at least 6 months following a positive case detection result to determine outcomes by Endocrinology Fellows. Adequate follow-up was defined as documentation of a positive case detection result in the EHR progress notes, ordering further evaluation (including repeat or confirmatory biochemical evaluation or imaging), or referral to subspecialties for further management. Patients who were deceased during chart review, and those who did not follow up with any primary care physician, nephrologist, cardiologist, or endocrinologist, were excluded. Our outcome measure considered referral to a specialist as adequate follow-up even if the visit was not completed yet at the time of analysis. Given the exploratory nature of this study and lack of prior data on follow-up rates after positive PA screening, formal power calculations were not performed. Descriptive statistics were obtained using the mean and standard deviation for continuous variables and frequencies and proportions for categorical variables. Differences between groups were assessed with Welch’s t-tests and Pearson’s Chi-squared test. The threshold of significance was P < 0.05. All statistical analyses were performed using R software version 4.3.0.

## Results

We identified 237 patients who met at least one of the three screening criteria between April 2018 and May 2023. The study population had a mean age of 60 years (SD 14), with a slight female predominance (51%, 120/237). The racial distribution was predominantly White (69%, 155/237), followed by Black or African American (26%, 58/237), and Asian (5%, 11/237). English was the primary language for 94% (224/237) of patients, while 6% (13/237) were non-English speakers.

Most patients had obesity (58%, 138/237) or were overweight (28%, 67/237), with only 13% (32/237) having normal BMI. Uncontrolled blood pressure (≥130/80 mmHg) was present in 85% (174/204) of patients with available data. Chronic kidney disease was documented in 26% (62/237) for stages 1-3a and 11% (25/237) for stages 3b-5/ESRD.

History of hypokalemia was present in 33% (78/237) of patients. Other comorbidities included obstructive sleep apnea in 22% (53/237) and prior hypertensive crisis in 14% (34/237). Mineralocorticoid receptor antagonist use was documented in only 8% (19/237) of patients at the time of screening. The different sample sizes across screening criteria (222, 237, and 180 patients respectively) reflect that individual patient could meet multiple criteria, with ARR ≥ 20 and PAC ≥ 10 ng/dL being the most inclusive criterion.

Among the 148 patients who received appropriate follow-up after positive screening, medical treatment emerged as the predominant approach, chosen for 66 (45%) patients. Another 32 (22%) patients were still undergoing additional diagnostic testing, while 22 (15%) patients had been referred to specialists. In 17 cases (12%), repeat or confirmatory testing yielded negative results. The remaining 11 (7%) patients were diagnosed with unilateral disease and either underwent adrenalectomy or had surgery planned.

Inadequate follow-up was observed across all screening criteria: 36.9% (82/222) of patients meeting PAC ≥ 10 ng/dL and PRA < 1 ng/mL/h, 37.6% (89/237) of those meeting ARR ≥ 20 and PAC ≥ 10 ng/dL, and 30% (53/180) of those meeting ARR ≥ 30 and PAC ≥ 10 ng/dL lacked appropriate care. Table 1 presents follow-up care rates among patients meeting different primary aldosteronism screening criteria and association between patient characteristics and adequate follow-up care across three primary aldosteronism screening criteria. Across all three screening criteria, history of hypokalemia (p < 0.001), English as primary language (p ≤ 0.007), and higher socioeconomic status (p ≤ 0.06) were consistently associated with receiving adequate follow-up care. Follow-up adequacy showed no significant differences based on age, gender, race, blood pressure control, chronic kidney disease stage, obstructive sleep apnea (OSA), and history of hypertensive emergencies, mineralocorticoid receptor antagonist (MRA) use or antihypertensive medication burden.

**Table 1:** Follow-up care rates and patient characteristics comparing adequate versus inadequate follow-up across three primary aldosteronism screening criteria.

Patient Characteristics	PAC ≥ 10 + PRA < 1			ARR ≥ 20 + PAC ≥ 10			ARR ≥ 30 + PAC ≥ 10		
	Adequate Follow Up N (%)			Adequate Follow Up N (%)			Adequate Follow Up N (%)		
	No	Yes	p-value^1^	No	Yes	p-value^1^	No	Yes	p-value^1^
	82 (37%)	140 (63%)		89 (38%)	148 (62%)		53 (30%)	127 (71%)	
**Age mean (SD)**	63 (14)	60 (13)	0.083	62 (15)	59 (13)	0.2	65 (12)	60 (13)	0.015
**Sex**			0.2			0.084			0.083
Female	47 (57%)	65 (46%)		52 (58%)	68 (46%)		32 (60%)	57 (45%)	
Male	35 (43%)	75 (54%)		37 (42%)	80 (54%)		21 (40%)	70 (55%)	
**Race**			0.8			0.8			0.3
Asian	5 (6.4%)	6 (4.5%)		5 (5.9%)	6 (4.3%)		4 (8.2%)	4 (3.4%)	
Black	20 (26%)	36 (27%)		21 (25%)	37 (27%)		15 (31%)	32 (27%)	
White	53 (68%)	90 (68%)		59 (69%)	96 (69%)		30 (61%)	82 (69%)	
Unknown	4	8		4	9		4	9	
**Language**			0.005			0.007			0.001
English	72 (88%)	137 (98%)		79 (89%)	145 (98%)		46 (87%)	126 (99%)	
Non-English	10 (12%)	3 (2%)		10 (11%)	3 (2%)		7 (13%)	1 (0.8%)	
**BMI**			0.088			0.05			0.036
Normal	15 (18%)	16 (11%)		16 (18%)	16 (11%)		11 (21%)	13 (10%)	
Obese	40 (49%)	89 (64%)		43 (48%)	95 (64%)		23 (43%)	80 (63%)	
Overweight	27 (33%)	35 (25%)		30 (34%)	37 (25%)		19 (36%)	34 (27%)	
**BP > 130/80**			>0.9			0.8			>0.9
No	11 (14%)	18 (16%)		11 (13%)	19 (16%)		8 (16%)	18 (17%)	
Yes	66 (86%)	97 (84%)		71 (87%)	103 (84%)		41 (84%)	87 (83%)	
Unknown	5	25		7	26		4	22	
**CKD stage 1, 2, 3a**			0.9			0.7			0.7
No	61 (74%)	107 (76%)		64 (72%)	111 (75%)		37 (70%)	94 (74%)	
Yes	21 (26%)	33 (24%)		25 (28%)	37 (25%)		16 (30%)	33 (26%)	
**CKD stage 3B, 4, ESRD**			>0.9			>0.9			0.8
No	74 (90%)	128 (91%)		80 (90%)	132 (89%)		46 (87%)	114 (90%)	
Yes	8 (10%)	12 (9%)		9 (10%)	16 (11%)		7 (13%)	13 (10%)	
**Hypokalemia**			<0.001			<0.001			<0.001
No	70 (85%)	78 (56%)		76 (85%)	83 (56%)		46 (87%)	64 (50%)	
Yes	12 (15%)	62 (44%)		13 (15%)	65 (44%)		7 (13%)	63 (50%)	
**OSA**			0.8			0.7			0.8
No	66 (80%)	107 (76%)		71 (80%)	113 (76%)		41 (77%)	94 (74%)	
Yes	16 (20%)	33 (24%)		18 (20%)	35 (24%)		12 (23%)	33 (26%)	
**Hypertensive crisis**			0.092			0.07			0.4
No	65 (79%)	124 (89%)		71 (80%)	132 (89%)		43 (81%)	111 (87%)	
Yes	17 (21%)	16 (11%)		18 (20%)	16 (11%)		10 (19%)	16 (13%)	
**MRA**			0.8			0.8			0.3
No	76 (93%)	127 (91%)		83 (93%)	135 (91%)		51 (96%)	115 (91%)	
Yes	6 (7%)	13 (9%)		6 (6.7%)	13 (8.8%)		2 (3.8%)	12 (9%)	
**>3 anti-HTN medications**			>0.9			>0.9			0.6
No	69 (84%)	116 (83%)		75 (84%)	124 (84%)		46 (87%)	105 (83%)	
Yes	13 (16%)	24 (17%)		14 (16%)	24 (16%)		7 (13%)	22 (17%)	
**Median Income**			0.04			0.06			0.022
1 (Extremely Low)	6 (7%)	1 (1%)		6 (6.7%)	1 (0.7%)		5 (9.4%)	1 (0.8%)	
2 (Very Low)	18 (22%)	41 (29%)		22 (25%)	44 (30%)		11 (21%)	37 (29%)	
3 (Low)	30 (37%)	53 (38%)		32 (36%)	53 (36%)		22 (42%)	48 (38%)	
4 (Moderate)	28 (34%)	44 (32%)		29 (33%)	49 (33%)		15 (28%)	40 (32%)	
Unknown	0	1		0	1		0	1	

^1^ Wilcoxon rank-sum test used for continuous variables. Chi-square tests used for categorical variables Continuous variables presented as mean (standard deviation); categorical variables as number (percentage). All analyses performed using R software version 4.3.0. P-values <0.05 considered statistically significant.

Abbreviations: PAC = Plasma Aldosterone Concentration (ng/dL); PRA = Plasma Renin Activity (ng/mL/h); ARR = Aldosterone-Renin Ratio; MRA = Mineralocorticoid Receptor Antagonist; CKD = Chronic Kidney Disease; ESRD = End-Stage Renal Disease; OSA = Obstructive Sleep Apnea; BP = Blood Pressure. Income Categories: 1 = Extremely Low Income (≤ $27,767); 2 = Very Low Income ($28,692 - $46,278); 3 = Low Income ($47,204 - $74,045); 4 = Moderate Income ($74,971 - $111,067).

## Discussion

This study evaluated follow-up rates of at least 6 months after a positive PA screening using 3 commonly applied case detection criteria: PAC ≥ 10 ng/dL and PRA < 1 ng/mL/h, (2) ARR ≥ 20 and PAC ≥ 10 ng/dL, and (3) ARR ≥ 30 and PAC ≥ 10 ng/dL. Approximately one-third of patients with a positive screening result did not receive follow-up interventions within this timeframe, regardless of the criteria used. This represents a substantial missed opportunity for early intervention in a condition where delayed diagnosis and treatment is associated with increased cardiovascular morbidity [9].

A study that surveyed practicing endocrinologists from Europe, Southeast Asia, Canada and Japan found that there is variability in the procedures for biochemical assessment of PA, the assays utilized, criteria to definite positive screening and criteria to forego confirmatory testing. While there were different criteria with different thresholds to define a positive screening, the combination of ARR and PAC was most common with the most common thresholds being utilized being 20 ng/dL/ng/mL/h for ARR and 10–15 ng/dL for PAC [12,18]. This variability in diagnostic approaches may contribute to inconsistent follow-up patterns, as clinicians may have different thresholds for acting on screening results based on their training and practice patterns. Our analysis of three common screening criteria revealed that follow-up patterns were remarkably consistent regardless of which threshold was applied. We initially hypothesized that stricter criteria (higher ARR values) might correlate with better follow-up, suggesting greater clinician confidence in these results. However, our findings indicate that clinical features such as hypokalemia had stronger associations with adequate follow-up than laboratory values. This highlights an important gap between laboratory measurements and clinical decision-making in PA evaluation that persists regardless of the specific ARR threshold applied.

Inadequate follow-up care after abnormal test results is not unique to PA [19–21]. Earlier studies on diabetes demonstrated that follow-up for abnormal results was frequently insufficient, even with targeted screening efforts [19–22].

Addressing these gaps requires systematic approaches. More recent research suggests that electronic medical records (EMRs) improve documentation and management of test results, including patient notification and follow-up [23]. Integrating standardized case detection criteria for PA into EMRs could address knowledge gaps in interpreting positive tests and ensure abnormal results are flagged for timely review by providers. These findings suggest that healthcare systems should prioritize developing standardized protocols and alert systems to ensure consistent follow-up of positive PA screening results. Potential automated alert models could include: (1) direct notifications to ordering providers when ARR values exceed institutional thresholds, (2) integration with existing clinical decision support tools that recommend mineralocorticoid receptor antagonist therapy for patients meeting specific criteria, or (3) automated referral generation to endocrinology for patients with severely elevated screening values. Such systems could be tailored to institutional workflows and provider preferences while maintaining standardized response protocols. Based on the findings from this study, our institution has since implemented automated electronic health record alerts for abnormal PA screening results to improve recognition and follow-up of positive cases. Future research should evaluate whether such system-level interventions improve follow-up rates and clinical outcomes.

Beyond automated alerts, addressing the knowledge gaps identified in our study requires comprehensive educational interventions. Clinician education through newsletters and continuing medical education should emphasize that hypokalemia occurs in only a minority of PA patients and should not be required for follow-up decisions. Undergraduate medical education curricula should incorporate updated PA diagnostic approaches to correct the traditional misconception that hypokalemia is prerequisite for PA investigation. These educational efforts could significantly improve recognition and appropriate follow-up of positive screening results.

Laboratory reporting systems should incorporate assay-specific cutoffs and interpretive comments with aldosterone and renin results. Given the variability in assays and reference ranges across institutions, standardized comments could guide clinicians on when further testing is indicated based on institution-specific thresholds. Such interpretive guidance, combined with the latest Endocrine Society guidelines for primary aldosteronism [24], (published after completion of our study period) could bridge the gap between laboratory results and clinical decision-making that our study identified.

The 33% prevalence of hypokalemia in our study is consistent with published literature reporting hypokalemia in 9–37% of PA patients [25], though our rate falls toward the higher end of this range. Patients with a history of hypokalemia demonstrated higher rates of follow-up care, likely reflecting its traditional recognition as a cardinal sign of PA [25–27]. However, this finding underscores a critical clinical misconception that may contribute to missed diagnoses. Normokalemia is present in the majority of PA cases, with hypokalemia occurring in only 9–37% of PA patients [25]. Therefore, the absence of hypokalemia should never dissuade clinicians from pursuing further investigation after positive screening. Clinicians must recognize that most patients with PA will have normal potassium levels, and follow-up decisions should be based on the positive screening results themselves rather than the presence or absence of hypokalemia.

The study also found that adequate follow-up was more common among English-speaking patients and those with higher median household income. It’s important to acknowledge that non-English speaking patients comprised only 10% of our study population, potentially limiting generalizability. Both language and income status may reflect broader disparities in healthcare access, though this relationship warrants further investigation in a more diverse patient cohort.

Mineralocorticoid receptor antagonist use was documented in only 8% (19/237) of patients at the time of screening, despite hypertension guidelines recommending MRA as a fourth-line medication in resistant hypertension. In addition, recent evidence indicates that MRAs may effectively reduce blood pressure and proteinuria in patients with suppressed renin activity (<1.0 ng/mL/h) and elevated aldosterone (≥4 ng/dL), even when these values fall outside traditional PA diagnostic criteria [28].

As an observational retrospective study using electronic health record data, our analysis has several limitations regarding continuity of care. While we defined adequate follow-up as occurring at least 6 months after screening, we could not determine the precise timing of follow-up appointments in relation to result availability. Although our institution’s electronic health record system requires providers to acknowledge laboratory results, we lacked data on the timeliness of result review or whether specific abnormal values triggered alerts to ordering providers. These workflow factors may have influenced follow-up patterns independently of the clinical characteristics we examined. Additionally, our composite definition of adequate follow-up, while clinically reasonable, was broad and may not capture the optimal sequence or specific quality of follow-up actions for individual patients.

This study represents the first systematic evaluation of follow-up patterns after positive PA screening. While our findings highlight important care gaps, the single-center design and reliance on electronic health record data limit generalizability. Multi-center studies and population-based analyses are needed to better characterize these care disparities and develop targeted interventions to improve PA management.

In summary, this study reveals a critical gap in the PA diagnostic pathway: approximately one-third of patients with positive screening results received no appropriate follow-up, representing missed opportunities for early intervention in a condition with significant cardiovascular morbidity. Our findings underscore the urgent need for comprehensive interventions including standardized protocols and automated decision support systems and targeted clinician education to ensure consistent follow-up after positive PA screening. Without addressing these multifaceted barriers, efforts to increase PA screening rates will fail to translate into improved patient outcomes.

## Abbreviations

PA: Primary Aldosteronism
BP: Blood pressure
BPA: Best Practice Alert
EHR: Electronic Health Record
ARR: Aldosterone to Renin Ratio
PAC: Plasma Aldosterone Concentration
MRA: Mineralocorticoid receptor antagonist
